# Obstructive Sleep Apnea in Psoriatic Arthritis: Clinical Characteristics and Comorbidities

**DOI:** 10.3390/biomedicines14030491

**Published:** 2026-02-24

**Authors:** Miguel A. Hernández-Mezquita, Esther Toledano, Rubén Queiró, Javier Martín-Vallejo, María José Fernández-Gómez, Carolina Cristina Chacón, Roberto Díaz-Peña, Pilar Sánchez-Conde, Daniel Martín, Cristina Hidalgo, María Dolores Sánchez, Inés Llamas-Ramos, Erik Díaz, Carlos Montilla

**Affiliations:** 1 Pneumology Department, Salamanca University Hospital, 37007 Salamanca, Spain; mhmezquita@gmail.com; 2 Department of Medicine, University of Salamanca (USAL), 37007 Salamanca, Spain; erik_diaz_1992@usal.es; 3 Institute of Biomedical Research of Salamanca (IBSAL), 37007 Salamanca, Spain; inesllamas@usal.es; 4 Department of Rheumatology, San Carlos Clinical Hospital, 28040 Madrid, Spain; esthertoledano@hotmail.com; 5 Rheumatology Department, Hospital Universitario Central de Asturias (HUCA), 33011 Oviedo, Spain; rubenque7@yahoo.es; 6 Statistics Department, Universidad de Salamanca, 37007 Salamanca, Spain; jmv@usal.es (J.M.-V.);; 7 Department of Rheumatology, Clinical University Hospital of Salamanca, 37007 Salamanca, Spain; ccchacon@saludcastillayleon.es (C.C.C.); chidalgoc15@gmail.com (C.H.); 8 Fundación Pública Galega de Medicina Xenómica, SERGAS, Grupo de Medicina Xenómica-USC, Health Research Institute of Santiago de Compostela (IDIS), 15700 Santiago de Compostela, Spain; roberto.diaz.pena@sergas.es; 9 Faculty of Health Sciences, Universidad Autónoma de Chile, Talca 3460000, Chile; 10 Department of Anesthesiology, Clinical University Hospital of Salamanca, 37007 Salamanca, Spain; pconde@usal.es; 11 Primary Care Management, Valladolid West, 47012 Valladolid, Spain; dmartinh96@gmail.com; 12 Rheumatology Department, Hospital de Zamora, 49021 Zamora, Spain; mariol333@hotmail.com; 13 Department of Nursing and Physiotherapy, Universidad de Salamanca, 37007 Salamanca, Spain; 14 Hospital of Salamanca, 37007 Salamanca, Spain

**Keywords:** obstructive sleep apnea, psoriatic arthritis, sleep-disordered breathing, pain amplification, non-inflammatory pain mechanisms, nociplastic pain

## Abstract

**Background**: Obstructive sleep apnea (OSA) is increasingly recognized in chronic inflammatory diseases, yet its prevalence and clinical correlates in psoriatic arthritis (PsA) remain poorly characterized. **Objective**: The objective of this study was to evaluate OSA prevalence and its relationship with disease activity, functional impairment, and comorbidities in PsA patients. **Methods**: A cross-sectional analysis of 247 consecutive PsA patients was conducted. OSA diagnosis was determined through medical record review. Disease activity was assessed using cDAPSA and ASDAS-CRP. Functional disability was measured using HAQ-DI and BASFI. Sleep quality (PSQI) and psychological symptoms (HADS) were evaluated. Inflammatory markers included CRP, IL-6, and TNF-α. Multivariable logistic regression identified independent predictors of OSA. **Results**: OSA prevalence was found to be 8.9% (22/247). OSA+ patients had significantly higher median age (58.0 vs. 54.0 years, *p* = 0.02), tender joint count (2.0 vs. 1.0, *p* = 0.002), functional disability (1.1 vs. 0.3, *p* = 0.001), fatigue (30.5 vs. 38.0, *p* = 0.04), anxiety (7.5 vs. 5.0, *p* = 0.03), depression (7.0 vs. 3.0, *p* = 0.004), and worse sleep quality (11.5 vs. 7.0, *p* = 0.001). Notably, no significant differences in inflammatory markers (CRP, swollen joints) were found between groups despite substantially higher pain burden in OSA+ patients. Female sex and greater tender joint count emerged as independent predictors of OSA. **Conclusions**: OSA occurs in ~9% of unselected PsA patients and is independently associated with functional disability, psychological distress, and elevated tender joint counts despite comparable inflammatory markers. This dissociation suggests that OSA drives pain amplification through non-inflammatory mechanisms. These findings support the use of systematic OSA screening in PsA patients with pain or disability disproportionate to inflammatory burden, particularly in those with psychological comorbidities.

## 1. Introduction

Psoriatic arthritis (PsA) is a chronic inflammatory arthropathy affecting up to 30% of patients with psoriasis, characterized by peripheral arthritis, axial involvement, and enthesitis [1,2]. Beyond articular manifestations, patients with PsA experience substantial disease burden associated with multiple comorbidities, including cardiovascular disease, metabolic syndrome, depression, and increasingly recognized sleep-related disorders [3,4].

Sleep disturbance in PsA has emerged as an important clinical concern, with prevalence rates varying from 30% to 85% depending on assessment methodology [5,6]. These sleep alterations are not merely secondary to articular pain or inflammation, but rather reflect complex changes in circadian regulation, central pain modulation, and intricate psychological factors [7]. Importantly, there is evidence that sleep quality and quantity are critical factors in the regulation of immune response, pain sensitivity, and psychological well-being in patients with chronic inflammatory diseases [8,9].

Obstructive sleep apnea (OSA), a sleep disorder, is a chronic condition in which the upper airway collapses intermittently during sleep, causing recurrent episodes of intermittent hypoxia and severe sleep fragmentation. Its prevalence ranges from 3% to 17% in the general population [10]. Recent evidence from a prospective polysomnography study in 42 patients with PsA reported an exceptionally high OSA point-prevalence of 50% in PsA patients, with 36% exhibiting moderate to severe disease, substantially exceeding general population estimates [11]. A study in a Danish cohort demonstrated an association between PsA and OSA, suggesting a shared pathophysiological mechanism [12,13]. This bidirectional relationship between PsA and OSA may result from chronic inflammation predisposing to upper airway dysfunction, while sleep fragmentation and oxygen desaturation perpetuate systemic inflammation [12,13]. Specifically, in patients with OSA, recurrent intermittent hypoxia activates hypoxia-inducible factor-1 alpha (HIF-1α) and nuclear factor kappa-B (NF-κB) signaling, resulting in the marked upregulation of pro-inflammatory cytokines including tumor necrosis factor alpha (TNF-α) and interleukin 6 (IL-6) [14]. Furthermore, these cytokines are associated with immune system activation and the perpetuation of articular inflammatory processes in PsA patients [15]. Despite this potential bidirectionality, few studies have systematically examined the relationship between PsA and OSA. Recent evidence indicates that OSA in PsA may operate through distinct pathophysiological mechanisms not fully explained by inflammatory markers. In a polysomnography-based study, no significant associations were identified between OSA occurrence and PsA disease activity assessed using disease activity indices (*p* > 0.05), despite elevated body mass index and advancing age as risk factors for OSA in this population [11]. In a Finnish cohort, pain intensity was associated with OSA presence, suggesting that OSA assessment could be important in the comprehensive management of PsA patients experiencing pain disproportionate to objectively measured inflammatory activity [16].

Given this background, we hypothesized that OSA prevalence in PsA has not been fully characterized in large cohorts, and the relationship between OSA and key PsA outcomes—including disease activity, functional impairment, disease impact, and comorbidities—remains unclear. Rather than focusing solely on prevalence estimates, this study emphasizes the clinical phenotype of PsA patients with OSA and the mechanisms through which sleep-disordered breathing may contribute to pain amplification independent of inflammatory burden.

## 2. Materials and Methods

This cross-sectional observational study was conducted at a single center from 7 January 2025 to 30 November 2025. Consecutive patients were recruited who met the following inclusion criteria:

(a) Diagnosed with PsA according to CASPAR criteria [15].
(b) Age ≥ 18 years.
(c) Provision of written informed consent.

The study protocol was approved by the Ethics Committee of the Hospital Universitario de Salamanca (CEIm Code: 2022 03 983-TFG, dated 2 June 2022). The study was conducted in accordance with the principles of the Declaration of Helsinki [16].

Patients were excluded if they were unable to adequately comprehend and complete the self-administered patient-reported outcome measures due to cognitive impairment or insufficient proficiency in the Spanish language.

### 2.1. Measured Variables

#### 2.1.1. Obstructive Sleep Apnea (OSA) Status

In this study, patients were classified as OSA+ if this diagnosis was documented in their clinical records (i.e., they had been previously diagnosed with OSA by polysomnography or sleep polygraphy, either attended or home-based). For all patients with confirmed OSA, the time elapsed since diagnosis was recorded (in years). Current treatment of patients diagnosed with OSA was also recorded. 

#### 2.1.2. Baseline Variables

The baseline variables were age (years), sex (coded as female = 0 and male = 1), disease duration (years), and disease phenotype, stratified as peripheral (pain and swelling in peripheral joints), axial (inflammatory back pain with radiographic sacroiliitis grade II or higher or syndesmophytes) [17,18], or mixed (meeting both criteria). Given the documented relationship between obstructive sleep apnea and cervical spine involvement in patients with spondyloarthritis, we evaluated the presence of radiographic abnormalities in PsA patients presenting with cervical pain and/or restricted cervical mobility [19]; smoking status (current smoker, former smoker, never smoked); treatment with conventional synthetic disease-modifying antirheumatic drugs (csDMARD), biologic disease-modifying antirheumatic drugs (bDMARD), or targeted synthetic disease-modifying antirheumatic drugs (tsDMARD); the number of affected entheses, measured using the modified Maastricht Ankylosing Spondylitis Enthesitis Scoring (mMASES) method [20]; and the presence of current or past dactylitis (yes/no). Skin disease severity was assessed using the Psoriasis Area and Severity Index (PASI), which evaluates the extent and severity of psoriatic lesions (erythema, induration, and desquamation) across four body regions (head, trunk, upper extremities, and lower extremities). Scores range from 0 to 72, with higher scores indicating greater severity [21].

#### 2.1.3. Disease Activity, Functionality, and Impact

For patients presenting with peripheral manifestations, disease activity assessment was accomplished using the Clinical Disease Activity Index for Psoriatic Arthritis (cDAPSA), a validated composite measure specific to PsA [22]. Notably, this clinical metric does not incorporate C-reactive protein values, distinguishing it from the full DAPSA. The cDAPSA composite score integrates tender joint count (TJC; range 0–68), swollen joint count (SJC; range 0–66), patient-reported global disease activity (numerical rating scale 0–10), and pain intensity (numerical rating scale 0–10)

In the case of patients with axial involvement, we used the Ankylosing Spondylitis Disease Activity Score with CRP (ASDAS-CRP) [23].

Functional ability was measured using the Health Assessment Questionnaire—Disability Index (HAQ-DI) for peripheral involvement [24] and the Bath Ankylosing Spondylitis Functional Index (BASFI) for axial involvement [25]. The HAQ-DI is a 20-question, self-administered questionnaire measuring disability in 8 functional categories [24]. Responses are scored from 0 to 3, where higher scores indicate greater disability. The BASFI is a 10-item patient-reported measure of spinal mobility and physical function in spondyloarthropathies, scored from 0 to 10, with higher scores indicating greater impairment [25].

Disease impact was assessed using the Psoriatic Arthritis Impact of Disease (PsAID-12) questionnaire [26], a 12-item patient-reported outcome measuring the impact of PsA on activities of daily living, symptoms, and emotional well-being.

#### 2.1.4. Comorbidities

Psychological comorbidities, specifically anxiety and depressive symptoms, were evaluated using the Hospital Anxiety and Depression Scale (HADS), a validated 14-item instrument for detecting affective disorders in medically ill populations. Raw scores for each domain (depression [HADS-D] and anxiety [HADS-A]) range from 0 to 21 and are stratified into interpretive categories: normal (0–7), borderline abnormality suggestive of possible psychopathology (8–10), or pathological range indicating probable clinical disorder (≥11) [27].

Fatigue was quantified using the Functional Assessment of Chronic Illness Therapy-Fatigue (FACIT-F) scale, a 13-item patient-reported measure with established psychometric properties in PsA populations. This instrument evaluates fatigue severity and its functional consequences across daily activities. The response format utilizes a 5-point Likert scale (0–4 per item), generating composite scores ranging from 0 to 52, where higher values reflect reduced fatigue severity [28].

Sleep quality was assessed using the Pittsburgh Sleep Quality Index (PSQI), a 19-item validated instrument capturing subjective sleep experience over the preceding month. This multidimensional measure evaluates seven components of sleep quality: perceived sleep quality, sleep latency, sleep duration, sleep efficiency, sleep disturbances, pharmacological sleep aids, and daytime somnolence. Each component receives domain-specific scoring (0–3), with summation yielding a global index (0–21), where higher scores denote greater sleep impairment. The clinical threshold for poor sleep quality is established at PSQI ≥ 6 [29].

Obesity was measured using the body mass index (BMI) [30].

Fibromyalgia was defined according to the 2016 revised American College of Rheumatology diagnostic criteria [31], which require the presence of widespread pain in ≥4 of 5 body regions combined with a Widespread Pain Index ≥ 7 and Symptom Severity score ≥ 5 (or WPI 4–6 and SS ≥ 9), sustained for ≥3 months.

#### 2.1.5. Analytical Variables

The analytical variables included C-reactive protein level (CRP) (mg/dL). In addition, given prior evidence indicating potential associations between sleep-disordered breathing and pro-inflammatory cytokine dysregulation, circulating levels of interleukin 6 (IL6) and tumor necrosis factor alpha (TNF-α) were measured using solid-phase immunometric assay technology (IMMULITE and IMMULITE 1000 automated systems, Siemens Healthcare Diagnostics, Llanberis, Wales, UK).

### 2.2. Statistical Analysis

Quantitative variables were expressed as means and standard deviations for normally distributed variables and as medians and interquartile ranges (IQR) for non-normally distributed variables. Qualitative variables were expressed as numbers and percentages (*n*/%). Normal distribution was assessed using the Shapiro–Wilk test.

Comparisons between two groups were performed using Student’s *t*-test for normally distributed quantitative variables and the Mann–Whitney U test for ordinal or non-normally distributed quantitative variables. Comparisons among more than two groups were performed using one-way ANOVA for normally distributed quantitative variables and the Kruskal–Wallis H test for ordinal or non-normally distributed quantitative variables.

Correlations between two quantitative variables were determined using Spearman’s rank correlation coefficient (rho), a non-parametric measure of monotonic correlation between variables.

A multivariable logistic regression analysis was conducted to explore the effect of variables that were significant (*p* < 0.05) in the univariate analyses or that had been described as relevant in the literature on the presence or absence of OSA [10,12,19,32]. The results are presented as odds ratios (OR) with 95% confidence intervals (95% CI). Model goodness-of-fit was assessed using the Nagelkerke R^2^ and the Hosmer-Lemeshow test. The variance inflation factor (VIF) has been calculated to explore possible effects of collinearity and associations between independent variables to explore possible confounding factors. To analyze the reliability and stability of the logistic regression results, the sample size was calculated based on the events per variable, the number of predictor variables, and the expected proportion. If 10 events per variable, 15 independent variables, and an expected proportion of 0.5 are established, the sample size would be 300 [33]. Statistical analyses were conducted using SPSS version 28 (IBM Corp, Armonk, NY, USA) for Windows. The “powermediation v. 0.3.4” function from R statistics v.4.5.0 was used to calculate the power of the model (R Foundation for Statistical Computing, Vienna, Austria).

## 3. Results

### 3.1. Demographic and Clinical Characteristics

Among the 247 PsA patients studied, 22 had confirmed obstructive sleep apnea (8.9%). The mean time since OSA diagnosis was 10.4 years (SD: 5). Sixteen patients (73%) presented moderate to severe OSA and the treatments they received were continuous positive airway pressure (CPAP) in 14 patients (64%) and other therapies such as mandibular advancement devices or surgical treatments in two patients (9%). In six patients (27%), only postural measures or dietary treatment were administered due to mild symptoms or an intolerance to the treatment with CPAP. The demographic characteristics and clinical parameters for the entire cohort and by OSA status are shown in Table 1.

### 3.2. Relationship of OSA with Activity, Functionality, and Impact

Patients with OSA exhibited a higher number of tender joints, worse functionality, and greater disease impact. The remaining variables are shown in Table 2.

### 3.3. Comorbidities

Patients with OSA had increased fatigue, higher anxiety (HADS-A) and depression (HADS-D) scores, worse sleep quality, and higher body mass index. The results are presented in Table 3.

### 3.4. Analytical Variables

No significant differences were found between the analytical variables. The results are presented in Table 4.

#### Multivariable Logistic Regression Analysis

Binary logistic regression analysis was performed with OSA presence/absence as the dependent variable and the following as independent variables: age, sex (coded as 0 = female, 1 = male), cervical involvement (coded as 0 = no, 1 = yes), smoking status, PASI, pain VAS, SJC, TJC, FACIT-F, HADS-A, HADS-D, PSQI, BMI, CRP, TNF-α, and IL-6.

In the logistic model, female sex (OR: 0.12; 95% CI: 0.02–0.63; *p* = 0.012) and greater TJC (OR: 1.6; 95% CI: 1.02–2.4; *p* = 0.037) emerged as independent predictors of OSA presence. The remaining variables did not achieve statistical significance: age (*p* = 0.070), cervical involvement (*p* = 0.971), smoking status (*p* = 0.618), PASI (*p* = 0.554), pain VAS (*p* = 0.209), SJC (*p* = 0.5), FACIT-F (*p* = 0.577), HADS-A (*p* = 0.311), HADS-D (*p* = 0.135), PSQI (*p* = 0.089), BMI (*p* = 0.438), CRP (*p* = 0.815), TNF-α (*p* = 0.834), and IL-6 (*p* = 0.828).

**Figure 1:**
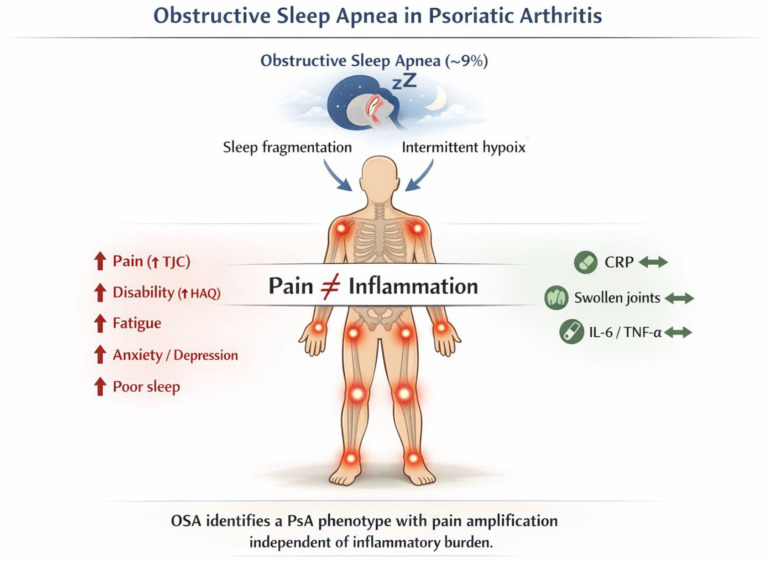
Graphical representation of the relevant findings.

The logistic model should be interpreted as exploratory due to the modest sample size of OSA+ patients (*n* = 22); however, the consistent association between TJC and OSA across both univariate and logistic model strengthens the hypothesis that this represents a clinically meaningful marker. The Nagelkerke R^2^ value of the model was 0.36 and the Hosmer-Lemeshow test was not statistically significant (Chi-square test = 9.688, df = 8, *p* = 0.289). No collinearity problems were detected, as all VIFs for the independent variables were close to one. The variable with the highest VIF was FACIT-F, with a value of 1.80. The main findings are shown in Figure 1.

## 4. Discussion

This study provides a comprehensive characterization of the prevalence and clinical correlates of OSA in psoriatic arthritis, demonstrating that approximately 8.9% of an unselected PsA cohort exhibited OSA. Rather than focusing primarily on the prevalence estimate, the key value of this investigation lies in its systematic characterization of the clinical phenotype associated with OSA in PsA: patients with comorbid OSA demonstrate a distinct clinical profile marked by pain amplification, functional disability, and psychological distress disproportionate to inflammatory burden. This phenotypic characterization identifies OSA-associated PsA as a clinically distinct subset warranting specific diagnostic attention and targeted therapeutic approaches.

The prevalence of OSA in this PsA cohort (8.9%) is lower than recently reported polysomnography (PSG)-based estimates (Mustafa et al., 50% in *n* = 42 PsA patients) [9] but higher than estimates reported for the general adult population, where OSA prevalence ranges from 3% to 17% depending on the diagnostic criteria applied [8,34,35,36]. This variance in prevalence estimates may be attributed to several factors, including methodological differences: while Mustafa et al. employed gold-standard polysomnography for OSA diagnosis in prospectively enrolled patients, our study relied on chart-based OSA diagnosis, which may potentially underestimate true prevalence compared to systematic PSG screening [9]. Nonetheless, both investigations consistently demonstrate that OSA prevalence in PsA substantially exceeds general population estimates, suggesting that the inflammatory and systemic nature of PsA predisposes patients to upper airway dysfunction beyond age-related and obesity-related factors alone.

In comparison to other chronic inflammatory arthropathies, a smaller study specifically examining sleep apnea prevalence across inflammatory arthropathies (RA, PsA, and peripheral spondyloarthritis) found an overall prevalence of 39.7% when combining both obstructive and central sleep apnea subtypes, with obstructive sleep apnea comprising approximately two-thirds of cases [37]. As previously discussed, these differences may be attributed to the methodology used in the definition of OSA.

Notably, in the Mustafa cohort, older age (OR = 1.17, 95% CI = 1.07–1.27, *p* = 0.001) and greater BMI (OR = 1.23, 95% CI = 0.99–1.52, *p* = 0.055) were identified as predictors of OSA in PsA [9], paralleling the association of age with OSA risk observed in general population studies. The difference in predictive variables between studies—with BMI and age in the Saudi cohort versus tender joint count in our cohort—suggests that disease-specific factors (articular pain signaling) may contribute to OSA risk in PsA alongside traditional obesity-related mechanisms.

Importantly, consistent with our findings, the Mustafa et al. study found no significant associations between OSA occurrence and PsA disease activity assessed by composite disease activity indices (DAPSA: 20.8 ± 12.4 in OSA+ vs. 15.7 ± 8.8 in OSA−, *p* = 0.3) [9]. Critically, both investigations demonstrate a striking dissociation between inflammatory markers and clinical symptoms in OSA+ patients: despite comparable CRP levels and swollen joint counts between groups, OSA+ patients experienced substantially greater TJC, suggesting that pain amplification operates through non-inflammatory mechanisms.

The association between OSA and increased pain burden observed in this cohort provides clinical evidence for a bidirectional relationship between sleep-disordered breathing and articular inflammation in PsA. On one hand, chronic peripheral inflammation characteristic of PsA may predispose to upper airway dysfunction through inflammatory infiltration of pharyngeal and laryngeal tissues, with the upregulation of pro-inflammatory cytokines promoting local edema and muscle dysfunction [38,39]. Conversely, and perhaps more compellingly, the intermittent hypoxia and sleep fragmentation inherent to OSA constitute potent drivers of systemic inflammation consistent with the growing recognition of OSA as a chronic low-grade systemic inflammatory condition [40]. Specifically, episodic hypoxia activates HIF-1α and NF-κB signaling cascades, resulting in the marked upregulation of pro-inflammatory cytokines including TNF-α and IL6 [12,41,42,43]. These same cytokines are central to the perpetuation of articular inflammation in PsA and other inflammatory arthropathies. Furthermore, these cytokines participate in the central amplification of chronic pain [44,45]. In this context, in our cohort, inflammatory markers (CRP: 0.2 mg/dL and SJC: 1.0 in both OSA+ and OSA− groups, *p* = 0.2 and *p* = 0.3, respectively) were not significantly elevated in OSA patients despite substantially greater pain burden (TJC 2.0 vs. 1.0, *p* = 0.002) and functional disability (HAQ-DI: 1.1 vs. 0.3, *p* = 0.001). This critical dissociation between objective inflammatory markers and clinical symptoms in OSA+ patients suggests that the pain amplification observed may operate through non-inflammatory mechanisms including sleep fragmentation, intermittent hypoxia-induced systemic inflammation, and impaired descending pain inhibition. Sleep fragmentation and chronic intermittent hypoxia are known to impair the descending pain inhibitory pathways through effects on monoaminergic neurotransmission (serotonin, noradrenaline, dopamine), potentially establishing or perpetuating central sensitization—a pain amplification phenomenon observed in approximately one-third of PsA patients [46,47]. Beyond sleep-disordered breathing per se, circadian disruption and poor light hygiene represent additional modifiable factors that may independently contribute to pain amplification and systemic inflammation in PsA. Dysregulated melatonin rhythms, irregular sleep–wake cycles, and nocturnal blue light exposure have been shown to upregulate IL-6/NF-κB signaling, thereby potentially sustaining the pro-inflammatory milieu that drives both articular symptoms and fatigue in these patients [48]. This broader neuroimmune-circadian framework extends the pathophysiological model from a purely anatomical perspective focused on upper airway obstruction to a more integrative paradigm encompassing chronobiological determinants of inflammation and pain. Thus, OSA may represent a potentially reversible driver of non-inflammatory pain in PsA patients who experience disproportionate symptom burden relative to their inflammatory burden, distinguishing this population from patients whose pain is primarily driven by inadequate inflammatory control.

The failure of BMI to predict OSA in multivariable logistic analysis in our cohort (*p* = 0.438), despite significant univariate association (*p* = 0.02), mirrors the pathophysiological complexity observed in the Mustafa cohort, wherein disease-specific mechanisms appear to supersede traditional obesity-related risk factors in determining OSA susceptibility in PsA. While obesity is a well-established risk factor for OSA in the general population and is associated with increased mechanical loading on upper airway structures and altered adipose tissue inflammation [49,50], the failure of BMI to remain an independent predictor in logistic regression model indicates that the association between tender joint count and OSA in this cohort reflects disease-specific factors—potentially including inflammatory cytokine upregulation, muscle dysfunction, and neuroimmune dysregulation—that contribute to OSA risk in PsA independently of traditional obesity-related mechanisms. The lack of statistical significance in the BMI model may also be due to the small sample size once the possible confounding effect of other variables included in the model was ruled out. The variable most strongly associated with BMI and OSA was gender, although these associations were weak and not statistically significant. In addition, the VIF value for BMI was 1.07.

In our study, as in the general population, sex was associated with the presence of OSA. In contrast to the findings of Mustafa et al., where sex was not a significant predictor of OSA (*p* = 0.17), in our study, female sex emerged as an independent predictor of OSA presence in multivariable logistic model (OR: 0.12; 95% CI: 0.02–0.63; *p* = 0.012), suggesting that women with PsA experience lower OSA risk than men. This association is consistent with epidemiological patterns observed in the general population, where previous studies have documented higher OSA prevalence in males [9,51].

The finding that OSA+ PsA patients experience greater pain despite comparable inflammatory markers, consistent across both our study and the Mustafa cohort, suggests that pain in these patients may represent a nociplastic rather than purely nociceptive or neuropathic pain phenotype [51,52,53]. Nociplastic pain, characterized by amplified pain signal processing in the absence of proportionate tissue pathology or evidence of active inflammation, may respond differently to conventional anti-inflammatory therapies compared to inflammatory pain. Thus, OSA screening may help identify a subset of PsA patients whose symptom burden reflects sleep-disordered breathing-induced pain amplification rather than therapeutic refractoriness to anti-inflammatory agents. From a practical standpoint, clinicians should consider OSA screening in PsA patients presenting with a specific phenotype: pain and functional disability disproportionate to objective inflammatory burden (low CRP, low SJC), elevated HAQ-DI despite adequate inflammatory control, poor sleep quality (PSQI ≥ 6), and psychological comorbidities (anxiety and/or depression). This constellation of findings may prompt respiratory medicine referral for OSA evaluation. Recognition of this phenotype is important because it suggests that therapeutic optimization may require both continued anti-inflammatory management and targeted OSA-directed interventions such as CPAP therapy or other sleep apnea treatments. The message that not all pain in PsA represents inflammatory disease activity requiring the escalation of anti-inflammatory therapy is clinically important and may guide more sophisticated, multidisciplinary treatment approaches aligned with emerging paradigms of “treat-to-target” strategies that incorporate both inflammatory and non-inflammatory pain mechanisms. CPAP therapy or other OSA-directed interventions might represent adjunctive approaches to improve pain control and functional outcomes in PsA patients with concurrent OSA, though prospective studies are needed to evaluate this hypothesis.

Several limitations merit acknowledgment. The cross-sectional design precludes causal inference regarding bidirectional relationships between OSA and PsA outcomes. The diagnosis of OSA was obtained through a review of medical records rather than a standardized and systematic polysomnography of all patients with PsA, which may underestimate the prevalence of OSA. Inflammatory markers (CRP, IL6, TNF-α) were measured at a single time point and may not reflect cumulative inflammatory burden, particularly in patients on disease-modifying therapy. Furthermore, circadian variation in inflammatory cytokines—particularly IL-6, which exhibits a well-documented diurnal rhythm with morning nadir and nocturnal peak [54] and potential differences in chronotype (habitual circadian phase preference) among participants may have introduced additional variability in inflammatory marker measurements. Although blood samples were collected within a standardized morning window (approximately 8:00–12:00 h), individual differences in circadian phase were not formally assessed, and future studies should incorporate chronotype evaluation to control for this potential source of bias. The relatively small absolute number of OSA+ patients (*n* = 22) limits statistical power for subgroup analyses and complex multivariable modeling. The logistic regression analysis should be interpreted as exploratory given this sample size. Finally, we did not collect data on OSA severity or treatment refractoriness. Although this information would complement the sample description, given the small number of patients with OSA (*n* = 22), stratified analyses by severity subgroups or treatment response would yield insufficient statistical power to draw meaningful conclusions.

However, this study has significant strengths. The sample size of 247 PsA patients represents one of the larger cohorts systematically evaluating OSA in PsA to our knowledge. The comprehensive clinical and psychosocial characterization, including anxiety/depression assessment, fatigue scales, and formal sleep quality measurement via PSQI, provides a more granular understanding of OSA-associated comorbidities in PsA than prior investigations. The comparison of inflammatory markers between OSA+ and OSA− groups, revealing no significant differences despite substantially greater pain and disability in OSA+ patients, provides direct clinical evidence for a non-inflammatory pain mechanism related to sleep-disordered breathing. Additionally, the systematic evaluation across multiple pain phenotypes (tender vs. swollen joints, global pain assessment) strengthens the characterization of pain in relation to OSA presence. The identification of TJC as an independent predictor of OSA presence in logistic regression analysis, rather than obesity-related factors, suggests that disease-specific inflammatory and neuroimmune mechanisms may contribute to OSA risk in PsA beyond traditional epidemiological risk factors.

## 5. Conclusions

Obstructive sleep apnea occurs in approximately 9% of unselected PsA patients and is substantially more prevalent than in the general population, although possibly lower than found in other chronic inflammatory joint diseases. OSA is independently associated with increased functional disability, fatigue, anxiety, and depression. Critically, OSA+ patients experience greater pain and functional impairment despite comparable inflammatory markers, suggesting that OSA may drive pain amplification through non-inflammatory mechanisms including sleep fragmentation, intermittent hypoxia-induced systemic inflammation, and impaired descending pain inhibition. The bidirectional relationship between systemic inflammation and sleep-disordered breathing in PsA implies that both inflammatory control and targeted OSA-directed interventions may be necessary to optimize symptom management in affected patients.

These findings support systematic OSA screening in PsA patients presenting with pain or disability disproportionate to objective inflammatory burden, particularly in those with elevated tender joint count and psychological comorbidities. A simple tool, such as the widely validated STOP-Bang test, can be very easy to apply for screening for OSA in these patients (Appendix A) [55]. Prospective studies evaluating whether CPAP therapy or other OSA interventions improve pain control and functional outcomes in PsA are warranted. Further investigation into the mechanisms linking intermittent hypoxia, systemic cytokine dysregulation, and pain amplification in the context of PsA may identify novel therapeutic targets for pain management in this disease and inform more sophisticated approaches to managing the multifaceted burden of OSA in inflammatory arthropathies.

## Figures and Tables

**Table 1:** Demographic and clinical characteristics stratified by obstructive sleep apnea (OSA) status.

Variable	All (*n* = 247)	OSA (*n* = 22)	NO OSA (*n* = 225)	*p*
Age	55 (14)	58 (18)	54 (14)	0.02
Sex, *n* (%)—female/male	109 (44.1)/138 (55.9)	6 (27.3)/16 (72.7)	103 (45.8)/122 (54.2)	0.09
Years since onset (years)	8 (8)	10 (7)	8 (8)	0.09
Smoking status *n* (%)				0.4
Smoker	72 (29)	4 (18)	68 (30)	
Former smoker	102 (41)	11 (50)	91 (41)	
Non-smoker	73 (30)	7 (32)	66 (29)	
Conventional synthetic DMARDs	146 (59)	16 (72)	130 (58)	0.1
Methotrexate	107 (73)	12 (76)	95 (73)	
Sulfasalazine	30 (21)	2 (12)	28 (22)	
Leflunomide	9 (6)	2 (12)	7 (5)	
tsDMARDs or bDMARDs, *n* (%)	64 (26)	6 (27)	58 (26)	0.8
TNF inhibitors	44 (69)	5 (83)	39 (67)	
IL17 inhibitors	7 (11)	0	7 (12)	
Ustekinumab	3 (4)	1 (17)	2 (3)	
Guselkumab	2 (3)	0	2 (3)	
iJAKs	3 (5)	0	3 (6)	
Apremilast	5 (8)	0	5 (9)	
Clinical presentation, *n* (%)				0.2
Peripheral	206 (82)	21 (92)	185 (72)	
Mixed	34 (15)	1 (8)	33 (22)	
Axial	7 (3)	0	7 (6)	
Cervical involvement (yes) (%)	8 (3.2)	1 (4.5)	7 (3.1)	0.5
Dactylitis (yes) (%)	40 (18)	5 (12)	35 (22)	0.07
mMASES	0 (2)	1.5 (3)	0 (2)	0.3
PASI	0.9 (2.2)	1.1 (2.4)	0.8 (2.2)	0.5

Data are presented as median (interquartile range) for continuous variables and *n* (%) for categorical variables, unless otherwise specified. Abbreviations: DMARD: disease-modifying antirheumatic drug; tsDMARD: targeted synthetic disease-modifying antirheumatic drug; bDMARD: biologics disease-modifying antirheumatic drug; mMASES: modified Maastricht Ankylosing Spondylitis Enthesitis Scoring; PASI: Psoriasis Area and Severity Index.

**Table 2:** Disease activity (peripheral and axial), functionality (peripheral and axial), and disease impact.

Variable	All (*n* = 247)	OSA (*n* = 22)	NO OSA (*n* = 225)	*p*
Pain VAS *	4 (4)	4 (4.2)	4 (4)	0.2
Activity VAS *	4 (4)	4.5 (4)	4 (4)	0.7
SJC *	1 (1)	1 (1)	1 (2)	0.3
TJC *	1 (2)	2 (2)	1 (2)	0.002
cDAPSA *	11 (8.7)	13.2 (9.2)	10.6 (9.3)	0.09
ASDAS-CRP **	2.3 (1.9)	2.4 (1.6)	2 (1.8)	0.6
HAQ-DI *	0.5 (1)	1.1 (0.9)	0.3 (0.8)	0.001
BASFI **	3.2 (6)	5.2 (6.2)	2.5 (4.3)	0.1
PsAID-12	3.2 (3.6)	3.8 (4.8)	3 (3.6)	0.04

Data are presented as median (interquartile range) for continuous variables and *n* (%) for categorical variables, unless otherwise specified. Abbreviations: VAS: visual analog scale; SJC: swollen joint count; TJC: tender joint count; cDAPSA: clinical Disease Activity Index for Psoriatic Arthritis; ASDAS-CRP: Ankylosing Spondylitis Disease Activity Score with C-reactive protein; HAQ: Health Assessment Questionnaire; BASFI: Bath Ankylosing Spondylitis Functional Index; PsAID: Psoriatic Arthritis Impact of Disease. * In peripheral and mixed forms (*n* = 240). ** In axial and mixed forms (*n* = 41).

**Table 3:** Comorbidities and psychological symptoms stratified by OSA status.

Variable	All (*n* = 247)	OSA (*n* = 22)	NO OSA (*n* = 225)	*p*
FACIT-F	38 (18)	30.5 (22)	38 (17)	0.04
HADS-A	5 (6)	7.5 (7)	5 (6)	0.03
HADS-D	3.5 (6)	7 (6.2)	3 (6)	0.004
PSQI	7 (7.5)	11.5 (12)	7 (8)	0.001
BMI (kg/m^2^)	27.5 (2.7)	29.7 (6.5)	25 (5.7)	0.02
Fibromyalgia (yes) (%)	14 (5.7)	2 (9.1)	12 (5.3)	0.4

Data are presented as median (interquartile range) for continuous variables and *n* (%) for categorical variables, unless otherwise specified. BMI is expressed as mean (standard deviation). Abbreviations: FACIT-F: Functional Assessment of Chronic Illness Therapy–Fatigue; HADS-A: Hospital Anxiety and Depression Scale—Anxiety; HADS-D: Hospital Anxiety and Depression Scale—Depression; PSQI: Pittsburgh Sleep Quality Index; BMI: body mass index.

**Table 4:** C-reactive protein and inflammatory cytokine levels by obstructive sleep apnea (OSA) status.

Variable	All (*n* = 247)	OSA (*n* = 22)	NO OSA (*n* = 225)	*p*
CRP (mg/dL)	0.2 (0.4)	0.2 (0.7)	0.2 (0.4)	0.2
IL6 (pg/mL)	2.7 (3)	3 (2.9)	2.7 (2.9)	0.1
TNF-α (pg/mL)	7.7 (5.2)	8.8 (3.9)	7.4 (5.5)	0.3

Data are presented as median (interquartile range) for continuous variables and *n* (%) for categorical variables, unless otherwise specified. Abbreviations: CRP: C-reactive protein; TNF-α: tumor necrosis factor alpha; IL-6: interleukin 6.

## Data Availability

The data presented in this study are available upon request from the corresponding author.

## Abbreviations

The following abbreviations are used in this manuscript:

OSA	Obstructive sleep apnea
PsA	Psoriatic arthritis
cDAPSA	Clinical Disease Activity Index for Psoriatic Arthritis
ASDAS-CRP	Ankylosing Spondylitis Disease Activity Score with C-reactive protein
HAQ-DI	Health Assessment Questionnaire—Disability Index
BASFI	Bath Ankylosing Spondylitis Functional Index
PSQI	Pittsburgh Sleep Quality Index
HADS	Hospital Anxiety and Depression Scale
CRP	C-reactive protein
IL-6	Interleukin 6
TNF-α	Tumor necrosis factor alpha
TJC	Tender joint count
SJC	Swollen joint count
csDMARD	Conventional synthetic disease-modifying antirheumatic drug
bDMARD	Biologic disease-modifying antirheumatic drug
tsDMARD	Targeted synthetic disease-modifying antirheumatic drug
mMASES	Modified Maastricht Ankylosing Spondylitis Enthesitis Scoring
PASI	Psoriasis Area and Severity Index
HIF-1α	Hypoxia-inducible factor-1 alpha
NF-κB	Nuclear factor kappa-B
PsAID-12	Psoriatic Arthritis Impact of Disease
FACIT	Functional Assessment of Chronic Illness Therapy
BMI	Body mass index
OR	Odds ratio
CPAP	Continuous positive airway pressure

## Appendix A. STOP-Bang Questionnaire

Instructions: Please answer Yes or No to each question.

**Table:**

Item	Question	Yes	No
S	Do you snore loudly (louder than talking or loud enough to be heard through closed doors)?	☐	☐
T	Do you often feel tired, fatigued, or sleepy during daytime?	☐	☐
O	Has anyone observed you stop breathing during your sleep?	☐	☐
P	Do you have or are you being treated for high blood pressure?	☐	☐
B	Is your body mass index (BMI) greater than 35 kg/m^2^?	☐	☐
A	Are you older than 50 years?	☐	☐
N	Is your neck circumference greater than 40 cm (16 inches)?	☐	☐
G	Gender: Male?	☐	☐
Scoring: Each “Yes” answer scores 1 point. 0–2: Low risk of obstructive sleep apnea ≥3: Intermediate to high risk of obstructive sleep apnea ≥5: High risk of obstructive sleep apnea			
